# Did School Meal Programs and SNAP Participation Improve Diet Quality of US Children from Low-Income Households: Evidence from NHANES 2013–2014?

**DOI:** 10.3390/nu13103574

**Published:** 2021-10-12

**Authors:** Tzuan A. Chen, Lorraine R. Reitzel, Ezemenari M. Obasi, Jayna M. Dave

**Affiliations:** 1 Department of Psychological, Health and Learning Sciences, College of Education, The University of Houston, 3657 Cullen Blvd. Stephen Power Farish Hall, Houston, TX 77204, USA; lrreitze@central.uh.edu (L.R.R.); emobasi@central.uh.edu (E.M.O.); 2 HEALTH Research Institute, The University of Houston, 4849 Calhoun Rd., Houston, TX 77204, USA; 3 USDA/ARS Children’s Nutrition Research Center, Department of Pediatrics, Baylor College of Medicine, 1100 Bates St., Houston, TX 77030, USA; jmdave@bcm.edu

**Keywords:** National Health and Nutrition Examination Survey (NHANES), Healthy Eating Index (HEI), school meals, Supplemental Nutrition Assistance Program (SNAP), children

## Abstract

Nutrition assistance programs such as school meals and the Supplemental Nutrition Assistance Program (SNAP) are designed to provide a safety net for the dietary intake of children from low-income families. However, compared with eligible non-participants, the relationship of diet quality with school meals only and school meals + SNAP is not well understood. The objectives of the study include: (1) To explore whether and to what extent nutrition assistance program participation (school meals only and school meals + SNAP) is related to diet quality; and (2) to examine the differences of diet quality between participating in school meals only, school meals + SNAP, or non-participation among American children. Children aged 5 to 18 years old from income eligible households who participated in the 2013–2014 National Health and Nutrition Examination Survey (NHANES) were included in this cross-sectional study (*n* = 1425). Diet quality was measured using the Healthy Eating Index (HEI)–2015 and its 13 subcomponents. A Rao-Scott Chi-square test, propensity scores approach, and Analysis of Covariance were performed. Covariates included age, sex, race/ethnicity, weight status, and family monthly poverty index. SAS survey procedures were used to incorporate the appropriate sample design weights. Participation in school meals + SNAP was not associated with higher diet quality compared to eligible non-participants or school meals-only participants. Participation in school meals + SNAP improved the intake of total dairy, but not added sugars or total vegetables compared to school meals only. Overall, school meal + SNAP participation did not significantly improve the overall diet quality of children in low-income households relative to comparable non-participants.

## 1. Introduction

Diet plays an important part in health [1,2]. Poor diet quality (e.g., high fats, added sugars, and low fiber) is a leading cause for chronic diseases like type 2 diabetes, cardiovascular disease, and some cancers such as breast cancer and colorectal cancer [3,4,5]. Furthermore, poor diet quality ranks among the top risk factors for chronic disease and mortality in the United States (US) [6]. The Healthy Eating Index–2015 was reported to be poor, just about 61–51 (out of 100) for US children (age 2–18), decreasing with age [7], implying poor adherence to US Dietary Guidelines among US children. Evidence showed that poor diet quality contributed to elevated disease risk among children such as hormone-related cancers and insulin resistance [8,9], and worse school performance [10]. In addition, children having poor diet quality are more susceptible to adverse health outcomes throughout life, as dietary behaviors developed in early life often carry into adulthood [9,11,12]. Multiple determinants, such as food preferences, health beliefs [13,14,15], socioeconomic status (SES) [16,17], and psychosocial precursors (e.g., lack of meal planning/shopping list) [18] are implicated in the increased rate of poor diet quality among the US population. SES is a complex construct, with years of education attainment and race/ethnicity as possible indicators [16,17]. A growing body of evidence suggests that SES and dietary intake are related, such that individuals from low-income families tend to have less capacity to adhere to the dietary guidelines due to limited access to healthy foods [16,17]. However, fewer differences in the adherence to dietary recommendations by income level were observed among children [17]. The household and school food environments, in particular, have a great influence on youths’ diet quality and behavior [19]. Diet during childhood is an important predictor of diet quality in adulthood [20], and thus, subsequent risk for diet-related morbidity. Thus, understanding the influences of adverse factors on the healthy dietary behavior of children is imperative, especially for those from low-income families who may have limited access and resources to secure healthy food.

Examination of a population’s dietary quality is critical because it provides guideline for public health policy. Individuals consume meals consisting of different foods that contain a combination of nutrients. Due to the complexity of dietary patterns, a composite measure summarizing the combinations of dietary components has been used to assess diet quality. One of the most used pre-existing diet quality indices in the US is the Healthy Eating Index (HEI) [21,22]. Poor diet quality is associated with elevated health risks that can impact costs of diet-related illness, such as diabetes, heart disease, and colorectal cancer—sizeable costs that are, in many cases, absorbed by the public [3,23,24]. Among children, there are many factors that may influence diet quality [19,25]. For example, one of the most important influences on the diet quality of children from low-income families is their participation in a nutrition assistance program [26,27,28].

Low-income households or individuals often rely on federal and community food and nutrition assistance programs to meet their dietary needs, and it is not unusual for eligible households to participate in more than one program [29]. Two of the largest federal nutrition assistance programs administered by the Food and Nutrition Service (FNS) of the US Department of Agriculture (USDA) are the Supplemental Nutrition Assistance Program (SNAP, formerly the Food Stamp Program) and the School Meal Programs including National School Lunch Program (NSLP) and School Breakfast Program (SBP) (hereafter, referred to as school meals).

SNAP is the largest federal food and nutrition assistance program through which eligible low-income households receive monthly benefits to purchase food at authorized retailers. In 2018, SNAP offered nutrition assistance to about 40.3 million individuals (20.1 million households) and provided an average monthly benefit of $126 per person ($254 per household) [30]. School meals provide meals for free or at a reduced price to children who are eligible, depending on family income guidelines. In 2019, about 29.6 million school children ate a NSLP meal each day, of which 74.1% were free or reduced-price meals [31], and 14.8 million children received a SBP meal each day, of which 85.1% were free or reduced-price meals [32]. All school meals are required to meet federal nutrition standards that align with the Dietary Guidelines for Americans (DGA) [33]. These school meals contribute a large portion of energy and nutrition for these children in low-income households [34].

Research assessing the relationship between nutrition assistance programs and diet quality have found mixed results [26,31,33]. For example, in one study, SNAP participants did not differ from low-income non-participants in daily caloric, macronutrient, and micronutrient intake [26]. However, SNAP participation increased certain HEI subcomponents scores (e.g., whole fruit) in adults [26,31], but not in children, as compared with low-income non-participants [26]. This is surprising as school meal programs, including the NSLP and the SBP, play an important role in children’s diets and can represent up to 58% of their daily food intake [34]. Additionally, the effects of SNAP participation on sugar-sweetened beverage consumption are also inconsistent [26].

Several studies have examined the relationship of participation in school meal program(s) and/or SNAP alone and diet quality among different populations [26,35,36,37,38,39]. However, we are unaware of studies that have specifically addressed the relationship between school meals, and school meals + SNAP combined (hereafter referred to as “nutrition assistance program participation,” for school meals, and/or school meals + SNAP combined) and diet quality. This study examined the association of nutrition assistance program participation with diet quality (as assessed by the Healthy Eating Index (HEI–2015)) among US children ages 5–18, using the 2013 to 2014 National Health and Nutrition Examination Survey (NHANES) data. The primary objectives were to identify: (1) whether participating in a nutrition assistance program (i.e., school meal only, and school meal + SNAP combined) was associated with diet quality, and (2) the differences in diet quality between school meals only, school meals + SNAP, and non-participation.

## 2. Materials and Methods

### 2.1. Participants

The study employed data from the NHANES 2013–2014. Secondary analysis of publicly available NHANES data was determined to be exempt from review by the University of Houston Institutional Review Board. NHANES is a cross-sectional, nationally representative survey of the civilian non-institutionalized population of the US conducted by the National Center for Health Statistics (NCHS) of the Centers for Disease Control and Prevention (CDC). Detailed information about each survey and its sampling design can be found elsewhere [21]. Children 5- to 18-years old with household income ≤200% of the federal poverty level (FPL) were included in the analyses. Participants with missing information on any of the main independent or dependent variables: SNAP participation, school meals participation, and HEI–2015 and its 13 subcomponents were excluded from the analysis. The final sample included 1425 children, with children categorized into three grade levels: elementary, middle, and high school. The NHANES protocols were approved by the National Center for Health Statistics Ethics Review Board of the U.S. CDC; written informed consent from all the participants was obtained prior to data collection.

### 2.2. Measures

#### 2.2.1. Diet Quality—Healthy Eating Index–2015

Diet quality was measured using the Healthy Eating Index (HEI–2015) [40], which assesses how well an individual’s dietary intake aligns with the Dietary Guidelines for Americans 2015–2020 [41]. The HEI–2015 was calculated using the simple HEI scoring algorithm for day 1 of the 24-h dietary recall data from the NHANES. Recall data were collected by trained interviewers using the USDA Automated Multiple Pass Method. Participants 11 years or younger were interviewed with assistance. Only weekday dietary data were used to assess the association of the HEI–2015 with the participation of school meals only and school meals + SNAP. In addition, only the day 1 recall was used because the day 2 recall had a higher rate of non-response and because people tended to report less consumption on day 2 (under-reporting or survey fatigue). The use of day 1 recall only has been used in previous published studies [34,42,43,44]. Additional information on the 24-h dietary recall procedure can be found elsewhere [45].

The HEI–2015 is a composite measure of 13 dietary elements with 9 adequacy components (i.e., Total Fruits, Whole Fruits, Total Vegetables, Greens and Beans, Whole Grains, Dairy, Total Protein Foods, Seafood and Plant Proteins, and Fatty Acids) and 4 moderation components (i.e., Refined Grains, Sodium, Added Sugars, and Saturated Fats). The details of the HEI–2015 have been reported elsewhere [21,46]. Scores on each component sum to have a maximum score of 100. For the adequacy components, higher scores reflect greater intakes. For the moderation components, higher scores reflect lower intakes because lower intakes are more desirable. For all components, a higher value indicates a better-quality diet. The higher the total score, the greater the dietary alignment with the Dietary Guidelines. An HEI score of >80 indicates a “good diet,” a score of 51–80 indicates a diet that “needs improvement,” and scores <51 reflect diet that is “poor” [46,47,48].

#### 2.2.2. Nutrition Assistance Program Participation

SNAP participation was assessed with the question “{Do you/Does any member of your household} currently receive SNAP or Food Stamp benefits?” Household level SNAP was used because it is possible that the respondents benefited directly or indirectly from household resource sharing even though they were not eligible for nutrition assistance. Households with low SES were eligible to participate in SNAP; moreover, if there were children in the households attending schools, they were also eligible to receive free or reduced-price school meals. Nevertheless, eligible individuals/households could decide whether or not to participate in each nutrition assistance program. Four nutrition assistance programs participation categories were identified to address this issue. First, nutrition assistance program participation was categorized as no participation or any participation. Subsequently, any participation was further categorized into school meals only (as school meals participation is the majority for single program participants), and school meals + SNAP combined.

#### 2.2.3. Covariates

Covariates included age, sex, race/ethnicity, weight status, and family monthly poverty index. Primary respondent’s age, sex, and race/ethnicity (Non-Hispanic White, Non-Hispanic Black, Hispanic, and Other Race) were reported in the NHANES Demographic Variables and Sample Weights Module, and family monthly poverty level index was included in the NHANES Income Questionnaire Module. Children’s body measurement data were collected by trained health technicians, and the Body Mass Index (BMI) was calculated using the standard formula (weight (kg)/height (m^2^)). Children’s weight status was classified into one of the three categories based on the CDC’s sex-specific 2000 BMI-for-age growth charts for the US: underweight and healthy weight (BMI < 85th percentile), overweight (BMI ≥ 85th percentile and BMI < 95th percentile), and obese (BMI ≥ 95th percentile) [49]. Due to infrequency of underweight category (2.69%), underweight and healthy weight were collapsed. The family monthly poverty level index, an index for the ratio of self-reported monthly income to poverty, was used as SES indicator in the analysis because it theoretically provides a comparable SES measure that can be applied across grade levels. It is an economic measure used to decide whether the income level of an individual or family qualifies them for certain federal benefits and programs. For example, to be able to receive reduced-price lunch at school. Moreover, income eligibility for participation in some federal programs, including reduced-price lunch, is that family monthly poverty level index should be less than 1.85 [50].

### 2.3. Statistical Analyses

To alleviate the issues of endogeneity and measurement error in observational studies, propensity scores were used to address the potential nonrandom self-selection bias [51].

The descriptive statistics of the participants’ characteristics were calculated for the three grade levels (elementary, middle, and high). The association of the participant characteristics and grade levels was tested using ANOVA or Rao-Scott Chi-square test for continuous and categorical variables, respectively. Sample-weighted data were used to account the NHNAES complex survey sampling methods such as survey non-response, and post-stratification adjustment. The descriptive statistics, ANOVA, and Rao-Scott Chi-square test were conducted using SAS 9.4 [52]. To address the potential self-selection bias among nutrition assistance program participants and eligible non-participants, propensity scores for the three grade levels were estimated using the mnps (multinomial propensity scores) function in the twang package [53,54,55] in R 3.5.0 [56]. The propensity score weighting method was applied to create two output datasets in which the distributions of the variables are balanced between groups. These two output datasets and groups include: (1) participation and non-participation, and (2) school meals only, school meals + SNAP, or non-participation. The procedure was performed using the covariates including age, sex, race/ethnicity, weight status, and family monthly poverty index as a set of potential confounding variables with potential correlations to nutrition assistance program(s) participation and diet quality. To compute the propensity scores through inverse probability of treatment weighting, these variables were used to fit a logistic regression model and a multinomial logistic regression model, using nutrition assistance program(s) participation and nutrition assistance program(s) non-participation, and school meals only, school meals + SNAP, or nutrition assistance program(s) non-participation as the outcome, respectively. The individual average treatment effect (ATE) weights were estimated through the covariates listed above, and the propensity scores were derived from the inverse probability of ATE weights. The calculated propensity scores were then incorporated into the final outcome models. The final outcome models were performed using SAS SURVEYREG procedure to account for the complex, stratified, multistage probability cluster sampling design. In the final outcome models, multiple linear regression models were used to evaluate the association between the two nutrition assistance program participation measures and the diet quality including HEI–2015 and its 13 subcomponents. The covariates included age, sex, race/ethnicity, weight status, and family monthly poverty index. The final outcome models were conducted separately for each outcome of interest (i.e., HEI–2015 and its 13 subcomponents) for the entire sample and by the three grade levels, and were performed using SAS 9.4 [52]. Statistical significance was set at *p* < 0.05.

## 3. Results

Among the 1425 schoolchildren included in this study, 48.17, 31.46, and 20.36% were elementary-, middle-, and high-school children, respectively (Table 1). The sex distribution was approximately equal (male: 49.22%, female: 50.78%), and the majority of the participants were non-Hispanic whites (41.55–45.67%) and healthy weight (56.52–61.31%) across each of the grade levels. The family monthly poverty level index ranged from 1.00 to 1.03 (below 1.85), implying meeting some federal assistance program eligibility and low SES among this analytic sample (Table 1).

The distribution of non-participation, participation in school meal program, and school meal program + SNAP varied by grade level (Table 1). The prevalence of the degree of the nutrition assistance program participation significantly differed by grade level (Rao-Scott χ^2^ = 40.81, *p* < 0.001). More than one-third of schoolchildren did not participate in school meals or SNAP; high-school children had the highest non-participation rate (54.64%). The higher the grade level, the lower rate the school meals + SNAP participation. In addition, BMI was significantly higher for more advanced grade levels/older children (*p*s < 0.001).

### Comparison between No Participation vs. Any Participation

The results showed that nutrition assistance program(s) participants had lower whole grains (4.26 ± 1.67 vs. 4.97 ± 1.71, *p* = 0.038) and higher total diary scores (7.22 ± 1.17 vs. 6.21 ± 1.19, *p* = 0.017) compared with eligible non-participants. There were significant differences in some of the HEI–2015 component scores by grade levels. Nutrition assistance program participants showed higher total dairy score (3.67 ± 5.00 vs. 2.22 ± 4.99, *p* = 0.037), but lower total fruits (−1.25± 3.75 vs. −0.12 ± 3.66, *p* = 0.026), whole fruits (−4.05 ± 3.76 vs. −2.54 ± 3.75, *p* = 0.01), and total HEI–2015 scores (21.45 ± 25.54 vs. 28.05 ± 25.22, *p* = 0.044) than non-participants in middle school children. There were no significant differences between participants and non-participants in elementary school and high school children (Table 2).

All of the groups, based on participation or grade levels, had poor diet quality (HEI–2015) ranging from 42.53 to 51.06. Children who participated in school meals + SNAP had higher total dairy scores (7.98 ± 0.51 vs. 6.95 ± 0.33, *p* = 0.049) and added sugars (5.47 ± 0.75, vs. 4.73 ± 0.76, *p* = 0.007) than non-participants and school meals-only participants, respectively. For elementary school children, school meals + SNAP participants had lower total vegetables scores compared with school meals-only participants (1.58 ± 0.31, vs. 1.96 ± 0.36, *p* = 0.03). School meals-only participants (6.81 ± 0.86 vs. 4.48 ± 0.67, *p* = 0.045) and school meals + SNAP participants (6.56 ± 0.84 vs. 4.48 ± 0.67, *p* = 0.029) consumed more sodium than eligible non-participants. School meals + SNAP participants also had higher refined grains scores than eligible non-participants (5.69 ± 0.74 vs. 3.75 ± 0.66, *p* = 0.028). For middle school children, non-participants had higher total fruits scores (2.00 ± 0.69, vs. −0.11 ± 0.60, *p* = 0.041), and whole fruits scores (2.09 ± 0.78, vs. −0.23 ± 0.50, *p* = 0.05) than school meals-only participants. For high school children, there was no significant difference found in eligible non-participants, school meals-only participants, and school meals + SNAP participants (Table 3).

## 4. Discussion

To our knowledge, this study is the first to investigate the association between diet quality and school meal program in conjunction with SNAP participation accounting for potential self-selection. School meal eligible children can receive a school breakfast/lunch free or at a reduced price from the NSLP and/or the SBP according to income guidelines. Children in households with incomes below 130% of the poverty level or those receiving SNAP qualify for free meals, and those with family incomes between 130–185% of the poverty line qualify for reduced-price meals [50]. Most of the eligible low-income families participated in more than one nutrition assistance program, which makes it challenging to measure the specific effect of any individual nutrition assistance program [57].

Several important findings have emerged from this study. Energy intakes were not different between participants and eligible non-participants across all three grade levels. Compared with eligible non-participants, participating in school meal program alone or school meals + SNAP combined was associated with fewer whole grains and more dairy consumption for all grade levels combined. Overall, children participating in nutrition assistance program(s) (school meals or school meals + SNAP) had lower HEI–2015 scores and energy intake relative to non-participants, although these differences did not reach statistical significance. These findings are partially consistent with previous research in a low-income sample of children focusing on single nutrition assistance program participation, where significant differences by SNAP participation were not found in total energy, HEI–2005 and macronutrients for children aged 4 to 19 [27] or in adults [26].

Among elementary school students in the current sample, students who only participated in school meals significantly consumed more total vegetables relative to those who participated in both school meals and SNAP. Although nutrition assistance programs aim to improve program participants’ dietary quality, our study showed that participation in nutrition assistance programs did not always help in reducing the intakes of HEI moderation components. Compared with eligible non-participants, the intake of sodium and refined grains was significantly higher for school meals alone participants or school meals + SNAP participants among elementary children. This finding complements and extends prior research on the effect of school meal programs on HEI moderation components. Excessive sodium and saturated fat consumption have also been shown to be associated with school meal program participation [38,58,59]. Those who were also receiving SNAP benefits consumed more added sugars and less total vegetables, which suggested that in addition to the school meals, household SNAP participation may only have a limited benefit to children’s diet. Previous research from the first School Nutrition Dietary Assessment Study (SNDA–I) found that NSLP participation led to a higher intake of dietary fat and a lower intake of added sugars than was found amongst non-participants [58]. Findings from SNDA-III showed both NSLP and SBP participation were associated with an increase in many key nutrients such as magnesium and vitamin C, but an increase in sodium was associated with NSLP participation alone [42].

It is notable that similar findings were found among middle school students. Our study revealed significant difference in HEI–2015 scores between non-participation and any nutrition assistance programs participation for middle school children, with nutrition assistance programs participants having significantly lower HEI–2015 relative to eligible non-participants; however, the difference of HEI–2015 scores between school meal program + SNAP participants and income-eligible nonparticipants was not significant. Previous studies have shown that around one out of five children in low-income households did not meet the dietary recommendations [27,60] and SNAP participation did not improve diet quality in children in low-income households [27].

Additionally, the present study also showed that the consumptions of total fruits and whole fruits were less for nutrition assistance program(s) participants (i.e., school meals only or school meals + SNAP) than eligible non-participants in middle schools. Further segregating the effects between school meals only and school meals + SNAP, the significantly lower total fruits consumption was only found for school meals-only participants relative to eligible non-participants. Lastly, in the current sample, no significant differences in HEI–2015 and subcomponents between nutrition assistance program participation and non-participation were found among high school students.

Although no prior studies showing participation in school meals was associated with decreased fruits consumption specifically using the sample of middle/high school students, previous research has revealed that overall diet quality was equivalent for NSLP participants and non-participants [61,62]. Additionally, there also have been undesirable associations observed between school meals and dietary measures such as fat intake [58,59]. Moreover, previous studies using NHANES data showed that SNAP participants had significant lower consumption of most components of diet including fruits relative to comparable low-income non-participants [26,63,64]. These findings suggest that nutrition assistance program participation may not improve the nutrition of children’s diet. To encourage the selection of vegetables and fruits, a prior study conducted in middle and high schools showed that school-level factors, such as a visual assessment of the quality of fruits and vegetables served and the variety of fruits and vegetables served, were associated with increased fruit and vegetable consumptions [65]. Available food resources within the home may fluctuate over time, as food stocks are usually replenished periodically [66]. Due to the variation of food stocks, the timing of the NHANES diet interview for participants may influence diet intake especially for participants from low-income households. Moreover, the use of SNAP might affect diet quality changes due to the timing of the survey, as other studies show that the SNAP cycle is relevant to the analysis [66]. Specifically, previous studies showed that SNAP participants experienced cyclic food intake with large declines near the end of the benefit month, implying the use of SNAP might affect diet quality changes due to the timing of survey or timing of the SNAP receipt [66].

There are several strengths and weaknesses of the current research that warrant discussion. The current study builds upon the extant literature regarding school-age children and nutrition assistance program participation with diet quality using the propensity score while adding to the literature by separating nutrition assistance programs’ effects that were not explored previously (school meals only or school meals + SNAP). The caloric intakes and HEI–2015 scores showed no significant differences among the degree of participation in nutrition assistance programs after adjusting for covariates and propensity score, meaning the participation in nutrition assistance program(s) did not improve participants’ diet quality relative to their nutrition assistance program-eligible non-participants.

The strengths of our study include the use of a large nationally representative sample of the US population to examine these associations. NHANES employs valid and reliable measure of diet, and standardized protocol for weight and height. Another strength of this study is the use of propensity scores to mitigate issues with endogeneity and self-section bias. The use of propensity scores has emerged as an approach in the estimation of a causal treatment effects in economic research. This approach is a statistical technique used in observational studies such as NHANES [67,68]; it accounts for unobserved individual differences when treatment assignment is not random. Given the nature of cross-sectional observational data like NHANES, nutrition assistance program participation might not directly affect the diet quality; yet, unobserved characteristics (e.g., health conditions) associated with nutrition assistance program participation might primarily account for the relationship with diet quality rather than participation itself. A previous study had shown that state SNAP policies and personal traits were strongly related to SNAP participation [69].

A limitation of this study was its cross-sectional nature, which does not allow causal inference. Similar to many previous studies, the results do not allow for interpretation of the causal effects of nutrition assistance program participation on diet outcomes. The lack of information on when the households were food insecure relative to when they received nutrition assistance program(s) benefits constrains the application of statistical analyses to address the self-selection bias issue on the nutrition assistance program participation [67]. Although our models adjusted for the possible confounding variables, and addressed self-selection bias with propensity scores, it is possible that unmeasured or unobserved confounding factors might affect both nutrition assistance program participation and diet quality. This may explain in part the association between nutrition assistance program participation and diet quality. Understanding of other unstudied factors, such as individual’s health status, is needed to optimize efforts to improve dietary quality for children participating in nutrition assistance programs. Single time-point assessments for dietary intake may be subject to day-to-day variation and underreporting is a common problem in dietary intake data. School meals and SNAP are included in this study to account for the interaction and scope of program participation for US youth but not the Special Supplemental Nutrition Program for Women, Infants9, and Children (WIC) due to the small number of WIC participants in this targeted population. Like previous studies, this study included weekday data only [34,70]. In addition, bias in diet assessment methods is possible due to the use of single dietary recall, and the potential measurement errors are associated with self-reported dietary intake. Another limitation is that this study did not examine sex, race, or weight status differences; however, they are worth exploring in the future. Although this study utilized data for 2013–2014, HEI−2015 was used to assess diet quality instead of HEI–2010. However, this is not a concern since several updates were made to NSLP and SBP meal guidelines in 2012 that align with HEI–2015 [71,72].

Findings from this study call for additional research to clarify the relationship of the nutrition assistance program participation with diet quality and to determine whether nutrition assistance program participation improves diet quality. Longitudinal studies are needed to address the issue of causality and elucidate the long-term effects of how the influence of nutrition assistance program participation changes with diet quality. Ultimately, if such studies find that nutrition assistance program participation enhances overall diet quality in low-income families, the potential health implications are substantial. Our results showed that participation in SNAP did not improve children’s diet quality; however, household-level SNAP participation was used in this study, so it is likely that NHANES participants did not benefit from the sources gained from other household members, as the benefit amounts might not be sufficient to smooth food intake over the benefit month for other household members. Furthermore, prior studies suggested that nutrition knowledge often associated with income and education level may partly mediate disparities in dietary intake and quality [64,73,74]. Therefore, the implementation of nutrition education programs alongside nutrition assistance programs is important for promoting awareness of healthy nutrition and improving health among low-income families, which could also serve as a prevention against increased disease risk in adult life [75].

## 5. Conclusions

Although nutrition assistance programs are aimed to provide a safety net for low-income households with children, school meals + SNAP participation did not improve the overall nutritional quality of children’s diets from low-income households. Participation in school meals + SNAP was not associated with better adequacy food components, higher diet quality, or lower moderation food components relative to eligible non-participants. SNAP participation along with school meals added the ameliorative effect on dairy but not added sugars and total vegetables except for within elementary school children. The current study was cross-sectional; however, the development of future longitudinal work is needed to delineate causal pathways between nutrition assistance program(s) participation and diet quality among children and adolescents to promote US youth’s health. Our findings highlight the need for additional research to better understand the complex interplay between nutrition assistance program participation and diet quality for youths from low-income families.

## Figures and Tables

**Table 1:** Descriptive Statistics of Participants Characteristics by Grade Level.

	All (*n* = 1425)	Elementary School (*n* ^‡^ = 761) ^a^	Middle School (*n* ^‡^ = 407) ^b^	High School (*n* ^‡^ = 257) ^c^
	Mean ± SD or *n* ^‡^(%)			
Age (years)	11.56 ± 0.16	7.95 ± 0.07	13.56 ± 0.05	17 ± 0.09
Sex				
Male	715 (49.22)	386 (48.67)	204 (49.52)	125 (50.04)
Female	710 (50.78)	375 (51.33)	203 (50.48)	132 (49.96)
Race/Ethnicity				
Hispanic	502 (28.25)	246 (27.51)	160 (29.71)	96 (27.77)
Non-Hispanic White	328 (44.35)	188 (45.62)	85 (41.55)	55 (45.67)
Non-Hispanic Black	415 (18.20)	239 (18.11)	108 (18.35)	68 (18.16)
Other Race	180 (9.20)	88 (8.76)	54 (10.39)	38 (8.41)
BMI (kg/m^2^) ^ab^***^, bc^***^, ac^***	21.49 ± 0.20	18.39 ± 0.22	23.24 ± 0.42	25.93 ± 0.6
Weight Status				
Underweight	35 (2.69)	21 (2.92)	6 (2.06)	8 (3.12)
Healthy Weight	785 (58.97)	427 (61.31)	220 (56.52)	138 (57.33)
Overweight	242 (17.82)	127 (17.56)	72 (19.98)	43 (15.15)
Obese	297 (20.52)	144 (18.22)	92 (21.45)	61 (24.40)
Family Monthly Poverty Level Index	1.01 ± 0.04	1.02 ± 0.04	1.00 ± 0.05	1.03 ± 0.08
Family Monthly Poverty Level Category				
Monthly Poverty Level Index ≤ 1.85	1223 (89.03)	663 (90.65)	347 (88.88)	213 (85.59)
Monthly Poverty Level Index > 1.85	100 (10.97)	44 (9.35)	34 (11.12)	22 (14.41)
Nutrition Assistance Program Participation ***				
No Participation	379 (35.63)	157 (29.15)	100 (33.24)	122 (54.64)
School Meals Only	538 (34.52)	293 (35.32)	156 (35.99)	89 (30.36)
School Meals + SNAP	508 (29.85)	311 (35.53)	151 (30.77)	46 (15.00)

Note: ANOVA and Rao-Scott Chi-square test were conducted to examine the participant characteristics differences among grade levels. ^‡^: unweighted sample size; ***: *p* < 0.001; BMI: body mass index; Mean and % are weighted using National Health and Nutrition Examination Survey exam weights. a, b, and c represent elementary school, middle school, and high school groups, respectively. ab: No Participation vs. School Meals Only; bc: School Meals Only vs. School Meals and SNAP; ac: No Participation vs. School Meals and SNAP.

**Table 2:** Mean Healthy Eating Index–2015 Components and Total Scores with Propensity Scores Adjusted as a Function of Any Nutrition Assistance Program Participation by Grade Level (*n* = 1425).

		No Participation	Any Participation	
		Mean ± SE		*p*-Value
All	Energy (kilocalories)	1825.05 ± 361.43	1821.28 ± 358.13	0.968
	Total Vegetables	0.66 ± 0.60	0.69 ± 0.60	0.920
	Greens and Beans	1.67 ± 0.79	1.5 ± 0.71	0.393
	Total Fruits	1.69 ± 0.87	1.37 ± 0.85	0.202
	Whole Fruits	1.65 ± 0.97	1.09 ± 0.95	0.079
	Whole Grains	4.97 ± 1.71	4.26 ± 1.67	**0.038**
	Dairy	6.21 ± 1.19	7.22 ± 1.17	**0.017**
	Total Protein Foods	3.53 ± 1.02	3.36 ± 0.97	0.236
	Seafood and Plant Proteins	3.14 ± 0.79	2.75 ± 0.81	0.102
	Fatty Acids	2.76 ± 1.57	2.1 ± 1.34	0.250
	Sodium	6.81 ± 1.75	6.82 ± 1.70	0.979
	Refined Grains	4.26 ± 1.68	4.34 ± 1.81	0.816
	Saturated Fats	6.54 ± 2.10	6.02 ± 1.99	0.297
	Added Sugars	4.18 ± 1.64	4.22 ± 1.63	0.920
	HEI–2015	48.07 ± 7.80	45.74 ± 7.56	0.237
Elementary School	Energy (kilocalories)	1031.13 ± 289.24	1005.36 ± 258.26	0.833
	Total Vegetables	1.32 ± 1.00	1.34 ± 0.91	0.924
	Greens and Beans	3.88 ± 1.12	3.5 ± 1.09	0.337
	Total Fruits	3.32 ± 1.29	3.36 ± 1.28	0.924
	Whole Fruits	3.86 ± 1.33	3.52 ± 1.18	0.519
	Whole Grains	7.06 ± 1.41	5.97 ± 1.47	0.083
	Dairy	4.17 ± 1.23	4.88 ± 1.29	0.253
	Total Protein Foods	4.94 ± 0.92	4.92 ± 0.82	0.942
	Seafood and Plant Proteins	6.82 ± 1.03	6.02 ± 1.09	0.052
	Fatty Acids	5.79 ± 1.53	5.51 ± 1.49	0.685
	Sodium	3.96 ± 1.38	4.34 ± 1.43	0.622
	Refined Grains	4.21 ± 2.96	5.09 ± 2.78	0.221
	Saturated Fats	8.16 ± 2.06	7.38 ± 1.96	0.235
	Added Sugars	7.19 ± 2.15	7.29 ± 2.08	0.834
	HEI–2015	64.67 ± 3.98	63.13 ± 4.38	0.538
Middle School	Energy (kilocalories)	2494.57 ± 1819.28	2451 ± 1726.53	0.809
	Total Vegetables	1.51 ± 6.33	1.73 ± 6.33	0.605
	Greens and Beans	6.06 ± 3.70	5.69 ± 3.72	0.069
	Total Fruits	−0.12 ± 3.66	−1.25 ± 3.75	**0.026**
	Whole Fruits	−2.54 ± 3.75	−4.05 ± 3.76	**0.010**
	Whole Grains	3.44 ± 6.72	2.86 ± 6.66	0.298
	Dairy	2.22 ± 4.99	3.67 ± 5.00	**0.037**
	Total Protein Foods	10.02 ± 4.67	9.6 ± 4.72	0.220
	Seafood and Plant Proteins	−0.86 ± 3.83	−1.29 ± 3.70	0.400
	Fatty Acids	0.75 ± 8.23	−0.1 ± 7.95	0.300
	Sodium	5.46 ± 8.59	4.55 ± 8.29	0.090
	Refined Grains	1.61 ± 8.63	0.66 ± 8.57	0.111
	Saturated Fats	6.78 ± 8.77	5.49 ± 8.92	0.143
	Added Sugars	−6.28 ± 4.73	−6.13 ± 4.70	0.824
	HEI–2015	28.05 ± 25.22	21.45 ± 25.54	**0.044**
High School	Energy (kilocalories)	842.26 ± 1720.47	1068.69 ± 1768.30	0.291
	Total Vegetables	−1.5 ± 2.19	−1.55 ± 2.29	0.886
	Greens and Beans	0.8 ± 2.01	0.9 ± 2.11	0.817
	Total Fruits	1.34 ± 3.63	1.54 ± 3.50	0.641
	Whole Fruits	4.44 ± 4.14	4.55 ± 4.02	0.837
	Whole Grains	9.85 ± 5.47	8.97 ± 5.47	0.277
	Dairy	−0.32 ± 3.46	0.99 ± 3.57	0.060
	Total Protein Foods	4.68 ± 3.12	4.38 ± 3.20	0.351
	Seafood and Plant Proteins	0.88 ± 3.13	0.77 ± 3.02	0.788
	Fatty Acids	9.04 ± 7.60	7.48 ± 7.68	0.074
	Sodium	4.97 ± 5.47	5.02 ± 5.44	0.951
	Refined Grains	14.22 ± 7.31	13.59 ± 7.19	0.447
	Saturated Fats	12.78 ± 7.11	12.13 ± 6.92	0.448
	Added Sugars	3.91 ± 6.81	4.75 ± 6.89	0.358
	HEI–2015	65.1 ± 24.68	63.51 ± 24.82	0.432

Note: The maximum score of HEI−2015 is 100. The 9 adequacy components are Total Vegetables, Greens and Beans, Total Fruits, Whole Fruits, Whole Grains, Dairy, Total Protein Foods, Seafood and Plant Proteins, and Fatty Acids; and the 4 moderation components include Sodium, Refined Grains, Saturated Fats, Added Sugars. ANOVAs were conducted to examine the Healthy Eating Index–2015 components and total scores Nutrition Assistance Program Participation between Nutrition Assistance Program Participation(s). Bold values denote statistical significance at the *p* < 0.05 level. 3.2. Comparison between no Participation, School Meals only, and School Meals + SNAP Combined.

**Table 3:** Mean Healthy Eating Index–2015 Components and Total Scores with Propensity Scores Adjusted as a Function of School Meals and SNAP Participation by Grade Level (*n* = 1425).

		No Participation ^a^	School MealsOnly ^b^	School Meals & SNAP ^c^	*p*-Value ^(ab)^	*p*-Value ^(ac)^	*p*-Value ^(bc)^
		Mean ± SE					
All	Energy (kilocalories)	1889.11 ± 74.56	1814.81 ± 135.02	1864.39 ± 156.76	0.873	0.585	0.608
	Total Vegetables	1.93 ± 0.17	1.80 ± 0.31	1.74 ± 0.28	0.644	0.756	0.685
	Greens and Beans	0.78 ± 0.19	1.01 ± 0.19	1.05 ± 0.22	0.336	0.270	0.839
	Total Fruits	2.54 ± 0.33	2.06 ± 0.40	2.12 ± 0.31	0.234	0.300	0.818
	Whole Fruits	2.43 ± 0.38	1.84 ± 0.29	1.81 ± 0.25	0.142	0.234	0.847
	Whole Grains	3.61 ± 0.43	3.04 ± 0.42	3.04 ± 0.44	0.386	0.235	0.999
	Dairy	6.95 ± 0.33	8.07 ± 0.54	7.98 ± 0.51	0.076	**0.049**	0.842
	Total Protein Foods	3.42 ± 0.19	3.12 ± 0.36	3.16 ± 0.38	0.332	0.302	0.832
	Seafood and Plant Proteins	1.92 ± 0.28	1.63 ± 0.40	1.77 ± 0.41	0.679	0.360	0.603
	Fatty Acids	3.70 ± 0.28	2.58 ± 0.52	3.27 ± 0.69	0.608	0.104	0.193
	Sodium	4.82 ± 0.47	5.82 ± 0.45	5.44 ± 0.53	0.419	0.146	0.148
	Refined Grains	5.33 ± 0.39	6.16 ± 0.41	5.95 ± 0.40	0.291	0.170	0.406
	Saturated Fats	5.69 ± 0.45	5.64 ± 0.59	5.81 ± 0.72	0.862	0.940	0.712
	Added Sugars	5.63 ± 0.52	4.73 ± 0.76	5.47 ± 0.75	0.825	0.189	**0.007**
	HEI–2015	48.74 ± 2.04	47.50 ± 1.71	48.60 ± 2.01	0.964	0.635	0.463
Elementary School	Energy (kilocalories)	1993.36 ± 44.31	1894.61 ± 126.51	1855.26 ± 124.52	0.347	0.536	0.748
	Total Vegetables	2.20 ± 0.25	1.96 ± 0.36	1.58 ± 0.31	0.173	0.589	**0.030**
	Greens and Beans	0.99 ± 0.23	1.68 ± 0.51	1.55 ± 0.40	0.210	0.206	0.536
	Total Fruits	3.10 ± 0.55	2.96 ± 0.55	3.12 ± 0.62	0.987	0.874	0.697
	Whole Fruits	2.93 ± 0.64	2.23 ± 0.41	2.11 ± 0.40	0.361	0.436	0.705
	Whole Grains	4.01 ± 0.46	3.03 ± 0.59	3.17 ± 0.65	0.332	0.175	0.789
	Dairy	7.51 ± 0.52	8.23 ± 0.71	8.1 ± 0.49	0.406	0.468	0.774
	Total Protein Foods	3.49 ± 0.29	3.14 ± 0.54	2.91 ± 0.64	0.418	0.570	0.388
	Seafood and Plant Proteins	2.35 ± 0.33	1.99 ± 0.62	2.12 ± 0.68	0.763	0.572	0.714
	Fatty Acids	3.54 ± 0.43	2.40 ± 0.74	2.93 ± 0.56	0.341	0.242	0.481
	Sodium	4.48 ± 0.67	6.81 ± 0.86	6.56 ± 0.84	**0.045**	**0.029**	0.574
	Refined Grains	3.75 ± 0.66	6.19 ± 0.77	5.69 ± 0.74	0.087	**0.028**	0.313
	Saturated Fats	6.67 ± 0.76	4.99 ± 0.84	4.95 ± 0.95	0.121	0.165	0.950
	Added Sugars	6.04 ± 0.64	4.83 ± 0.64	4.88 ± 0.71	0.164	0.202	0.892
	HEI–2015	51.06 ± 2.92	50.44 ± 3.44	49.65 ± 3.65	0.775	0.901	0.717
Middle School	Energy (kilocalories)	1690.78 ± 139.89	1593.19 ± 248.52	1476.52 ± 213.00	0.519	0.789	0.356
	Total Vegetables	2.00 ± 0.45	2.35 ± 0.74	2.30 ± 0.67	0.743	0.713	0.894
	Greens and Beans	0.81 ± 0.37	0.90 ± 0.80	0.87 ± 0.84	0.945	0.919	0.926
	Total Fruits	2.00 ± 0.69	−0.11 ± 0.60	−0.16 ± 0.50	**0.041**	0.071	0.899
	Whole Fruits	2.09 ± 0.78	−0.23 ± 0.50	−0.35 ± 0.69	**0.050**	**0.050**	0.749
	Whole Grains	3.34 ± 1.44	0.87 ± 1.50	0.74 ± 1.49	0.315	0.334	0.786
	Dairy	6.41 ± 0.88	7.77 ± 0.72	7.86 ± 0.87	0.364	0.284	0.896
	Total Protein Foods	2.97 ± 0.41	3.32 ± 0.74	3.63 ± 0.73	0.363	0.647	0.459
	Seafood and Plant Proteins	1.83 ± 0.62	0.76 ± 0.71	0.74 ± 0.87	0.390	0.321	0.966
	Fatty Acids	2.92 ± 0.67	4.06 ± 1.13	4.48 ± 1.21	0.239	0.395	0.547
	Sodium	4.96 ± 0.64	3.07 ± 1.06	2.94 ± 1.27	0.107	0.127	0.845
	Refined Grains	6.30 ± 1.08	7.79 ± 1.16	8.04 ± 1.05	0.358	0.448	0.654
	Saturated Fats	4.42 ± 1.02	5.56 ± 1.28	5.39 ± 1.03	0.539	0.563	0.810
	Added Sugars	6.09 ± 0.95	4.89 ± 1.34	6.06 ± 1.57	0.984	0.380	0.085
	HEI–2015	46.15 ± 5.87	41.00 ± 4.87	42.53 ± 5.78	0.706	0.580	0.554
High School	Energy (kilocalories)	1747.88 ± 183.81	1673.46 ± 443.44	2306.88 ± 241.49	0.061	0.858	0.069
	Total Vegetables	1.69 ± 0.26	1.54 ± 0.63	1.84 ± 0.49	0.807	0.789	0.660
	Greens and Beans	0.40± 0.34	0.86 ± 0.61	1.33 ± 0.43	0.120	0.511	0.401
	Total Fruits	1.64 ± 0.40	2.31 ± 0.72	1.60 ± 0.50	0.946	0.346	0.077
	Whole Fruits	1.85 ± 0.54	2.05 ± 0.80	1.81 ± 0.59	0.944	0.817	0.609
	Whole Grains	3.26 ± 0.84	3.24 ± 1.09	2.31 ± 1.05	0.408	0.983	0.263
	Dairy	6.83 ± 0.58	8.97 ± 1.41	8.23 ± 1.25	0.265	0.114	0.468
	Total Protein Foods	3.44 ± 0.24	3.10 ± 0.53	2.84 ± 0.37	0.181	0.560	0.604
	Seafood and Plant Proteins	1.00 ± 0.46	1.10 ± 0.41	1.05 ± 0.56	0.935	0.851	0.943
	Fatty Acids	4.51 ± 0.78	1.89 ± 1.26	3.16 ± 1.48	0.379	0.063	0.292
	Sodium	4.30 ± 0.80	4.88 ± 1.11	3.89 ± 0.87	0.682	0.671	0.279
	Refined Grains	5.97 ± 0.83	5.91 ± 0.91	5.42 ± 0.77	0.637	0.960	0.407
	Saturated Fats	5.78 ± 0.99	5.05 ± 1.52	5.05 ± 1.63	0.564	0.592	0.995
	Added Sugars	4.72 ± 0.91	5.28 ± 2.05	6.54 ± 1.53	0.136	0.741	0.190
	HEI–2015	45.39 ± 2.17	46.17 ± 5.24	45.05 ± 4.46	0.930	0.883	0.768

Note: The maximum score of HEI−2015 is 100. The 9 adequacy components are Total Vegetables, Greens and Beans, Total Fruits, Whole Fruits, Whole Grains, Dairy, Total Protein Foods, Seafood and Plant Proteins, and Fatty Acids; and the 4 moderation components include Sodium, Refined Grains, Saturated Fats, Added Sugars. ANOVAs were conducted to examine the Healthy Eating Index–2015 components and total scores Nutrition Assistance Program Participation among School Meals and SNAP Participation. Bold values denote statistical significance at the *p* < 0.05 level. Letter a, b, and c represent No Participation, School Meals Only, and School Meals and SNAP, respectively. ab: No Participation vs. School Meals Only; bc: School Meals Only vs. School Meals and SNAP; ac: No Participation vs. School Meals and SNAP.

## Data Availability

The data analysis used publicly available data which can be accessed at: https://wwwn.cdc.gov/nchs/nhanes/continuousnhanes/default.aspx?BeginYear=2013 (accessed on 31 January 2019).
